# Full-dose intra-operative radiotherapy with electrons (ELIOT) during breast-conserving surgery: experience with 1246 cases

**DOI:** 10.3332/eCMS.2008.65

**Published:** 2008-02-26

**Authors:** U Veronesi, R Orecchia, A Luini, V Galimberti, G Gatti, M Intra, P Veronesi, MC Leonardi, M Ciocca, R Lazzari, P Caldarella, N Rotmensz, C Sangalli, LS Silva, D Sances

**Affiliations:** 1 Scientific Director, European Institute of Oncology, Milan, Italy; 2 Division of Radiotherapy, European Institute of Oncology, Milan, Italy; 3 Division of Breast Surgery, European Institute of Oncology, Milan, Italy; 4 Physics Unit, European Institute of Oncology, Milan, Italy; 5 Division of Epidemiology and Biostatistics, European Institute of Oncology, Milan, Italy; 6 Division of Anesthesiology, European Institute of Oncology, Milan, Italy; 7 University of Milan, Italy; 8 Postgraduate School of General Surgery, University of Perugia, Italy

## Abstract

**Background:**

Previous studies showed that after breast-conserving surgery for breast cancer, radiotherapy may be applied to the portion of the breast where the primary tumour was removed (partial breast irradiation (PBI), avoiding the irradiation of the whole breast. We developed a procedure of PBI consisting of a single high dose of radiotherapy of 21 Gy with electrons equivalent to 58–60 Gy in fractionated doses, delivered during the surgical session by a mobile linear accelerator, positioned close to the operating table.

**Patients and methods:**

From July 1999 to December 2006, 1246 patients with primary carcinoma of less than 2.5-cm maximum diameter, mostly over 48 years, were treated with electron intra-operative radiotherapy (ELIOT) at a single dose of 21 Gy.

**Results:**

After a follow-up from 0.3 to 94.7 months (median 26), 24 (1.9%) patients showed a local recurrence and 22 developed distant metastases. Sixteen patients died, seven from breast carcinoma and nine from others causes. The five-year crude survival was 96.5%. Six (0.5%) developed severe breast fibrosis, which resolved in 2–3 years. An additional 40 patients suffered for mild fibrosis. Cosmetic results were good.

**Conclusions:**

Electron intra-operative radiotherapy is a safe method for treating conservatively operated breasts and avoids the long period of post-operative radiotherapy, greatly improving the quality of life and reduces the cost of radiotherapy. ELIOT markedly reduces the radiation to normal surrounding tissues and deep organs. Results on short- and medium-term toxicity are good. Data on local control are encouraging.

## Introduction

Partial breast irradiation (PBI) after conservative surgery, the pin-point use of radiation fields on a limited involved portion of the breast rather than the whole organ, is a procedure that is gaining ground in breast cancer treatment with many studies showing encouraging results [1–4]. Another important theme under discussion is intra-operative radiotherapy (IORT), which has been utilized in the last few decades to irradiate, with an external single dose, different types of tumours, mainly in the abdominal cavity during surgery [5]. The limitation of its use, due to the need to transfer the patient from the surgical theatre to the radiotherapy department during surgery, was recently overcome by the availability of mobile linear accelerators, which can enter the surgical theatre.

In 1999 we developed the idea of combining the two procedures (PBI and IORT) to irradiate the breast during conserving surgery in a single session, and we started a series of clinical studies whose preliminary results were previously reported. With the new technique, which we defined electron intra-operative therapy (ELIOT), the mobile linear accelerator delivers a single dose of radiation with electrons to the involved quadrant of the breast during surgery, shortening the radiotherapy course from six weeks to one single session during surgery [6–9].

We describe here the results from a series of 1246 breast cancer patients treated with ELIOT from 1999 to 2006.

## Patients and methods

From July 1999 to December 2006, 1246 breast cancer patients (mean age 57 years, range 33–80) were treated with ELIOT after breast-conserving surgery. All patients had unicentric primary carcinoma, with a maximum largest diameter of 2.5 cm on clinical or radiological examination. Before reaching the full dose of 21 Gy, 33 patients were treated with ELIOT at various doses, progressively ranging from 10 to 19 Gy as part of the initial dose-finding study. The remaining 1213 patients received 21 Gy prescribed at the 90% isodose as sole radiation treatment. Most of the patients were over 48 years, due to the inclusion criteria we established for our protocol. As we are conducting ‘separate’ a randomized trial comparing ELIOT single dose with traditional external radiotherapy, all eligible patients were normally asked to participate in that trial. The patients described in the present paper are those that either did not chose to enter the randomized trial or did not fulfil the eligibility criteria. They are many in number, but this reflects the fact that more than 3000 patients are operated each year in IEO for breast cancer. The randomized trial criteria were tightly observed when treating these patients. Many of the excluded patients requested to be treated by the ELIOT technique. These patients were clearly advised that the procedure was experimental, and they then signed an informed consent form, in which they acknowledged their understanding of the experimental nature of their treatment. The characteristics of the patient population are summarized in Tables 1 and 2. Patients who declined to sign the informed consent form were treated with standard post-operative whole breast irradiation over six weeks.

## Surgery

As regards surgery, 1125 patients received quadrantectomy with axillary sentinel node biopsy; of these, 888 (71.3%) had a negative sentinel node and so axillary dissection was not performed, 358 (28.7%) showed a metastatic sentinel node and 311 of these received complete axillary dissection, while 47 patients with micro-metastatic involvement, being part of a randomized study, did not undergo axillary dissection. Eighty-five patients received quadrantectomy with immediate complete axillary dissection due to clinically positive nodes. Thirty-two patients received simple quadrantectomy, either because the carcinoma was minimal or due to difficult general conditions. Four cases had other forms of local treatment.

## Operative technique

### Tumour removal

According to the tumour site and size and breast size and shape, wide breast resection was done according to the standard quadrantectomy. The ELIOT procedure does not interfere with the oncologic criteria of ‘classic’ breast-conserving surgery (BCS) in which 1.5–2 cm grossly free margins of resection are required.

After the tumour removal, the wide mobilization of the mammary gland from the fascia of the pectoralis major and, superficially, from the skin, represents a critical step, permitting the optimal exposure of the ‘target’ to the radiation beam. The deep surface of the breast is easily mobilized from the fascia of the pectoralis major muscle for at least 4–5 cm around the tumour bed to approximate the parenchyma of the central area and to expose the mammary parenchyma to the radiation beam. The superficial margins of the parenchyma must also be carefully separated from the subcutaneous tissue at the level of the anterior adipose lamina for 3–5 cm in every direction.

### Thoracic wall protection

To minimize the radiation delivered to the thoracic wall and to guarantee the delivery of the full radiation dose to the gland, a dedicated disk of lead and aluminium, available in various diameters (4–6 cm), is used as a protective device. The disk is inserted (lead down, aluminium up) in the space between the gland and the pectoralis muscle. To allow the best protection of the thoracic wall, the disk must be greater in size than the breast target size. According to the extent of the skin incision and to the anatomical local conditions whenever possible, the largest possible disk is placed exactly under the mammary target to be irradiated.

## Breast gland reconstruction

The gland must be reconstructed over the disks to expose the correct portion of the breast to the radiation beam, thereby avoiding excessive inhomogeneity in the shape of the target volume. The electron beam energy is chosen on the basis of the thickness of the target volume, and the best dose distribution of radiotherapy in the gland is achieved if the thickness of the irradiated target remains as homogeneous as possible. The gland thickness is then measured using a simple needle (perpendicularly inserted through the breast target until the hard surface of the disk can be felt), and the correct electron energy is selected. The exact site where the tumour was located is marked by ink on the mammary gland surface. This point represents the centre of the target for the irradiation.

### IORT collimator placement and connection to the linear accelerator

The sterile polymethyl methacrylate (Perspex; Hitesys SpA, Aprilia, Italy) collimator of the linear accelerator (LINAC) is introduced through the skin incision and placed directly in contact with the breast target. The collimator is centred on the previous ink marker on the mammary gland surface. Correct positioning of the collimator is fundamental to guarantee the coverage of the entire target volume.

The portion of the breast that needs to be irradiated (clinical target volume) is generally an area of 4–6 cm of diameter around the cancer resection site. The collimator diameter is selected on the basis of the choice of the area to be treated to ensure the adequacy of the coverage of the entire tumour bed plus a safe margin. The collimator is placed directly in contact with the breast gland, after moving the LINAC by remote control to reach the operating table.

## Radiotherapy

**Figure 1:**
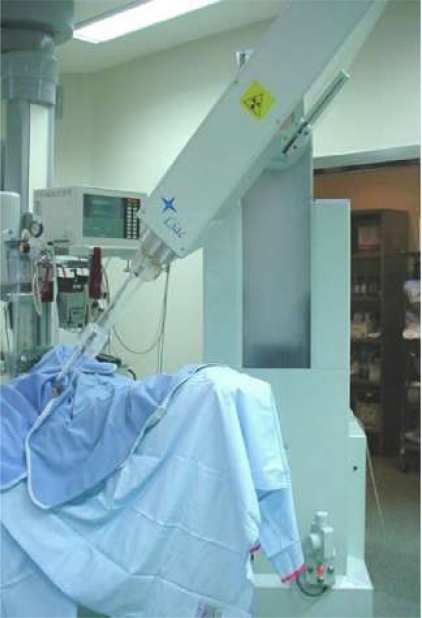
The linac used for ELIOT

Electron intra-operative radiotherapy technique has been described in previous papers [6–9]. Briefly, two mobile linear accelerators (Figure 1) were used to deliver electrons, a Novac7 (Hitesys Srl, Latina, Italy) and a Liac (Info&Tech, Roma, Italy), installed in two different operating rooms. The two linear accelerators, which can be easily manoeuvred by means of motors acting on the wheels and on the robotic arm, deliver electrons at the following different nominal energies: 3, 5, 7 and 9 MeV (Novac7) and 4, 6, 8 and 10 MeV (Liac). The collimation of the beam is achieved by a hard-docking system, consisting of perspex applicators, 5-mm thick. The flat-ended and bevelled (15° up to 45°) round applicators have a diameter ranging from 4 to 12 cm. The nominal source to surface distance (SSD) is 80–100 cm for Novac7 and 60 cm for Liac. The radiation protection is obtained by a primary beam stopper, consisting of a trolley-mounted 1.5-cm-thick lead shield and some mobile 1.5-cm-thick lead shielding (100 cm long, 150 cm high).

In the Quality Assurance programme for those dedicated linear accelerators, we have implemented an *in vivo* dosimetry procedure, aimed at controlling the dose delivered to the patient [10].

Every patient was evaluated one, three, six and 12 months after surgery, and thereafter every six months, to look for early, intermediate and late complications. The follow-ups ranged from 0.3 to 92 months (mean 26.2). Twelve patients were lost to follow-up.

## Adjuvant treatments

Adjuvant treatments were given according to the rules and protocols in force during the period of patient accrual at the European Institute of Oncology.

Eight-hundred-and-seven patients received endocrine treatment, 128 patients were treated with chemotherapy, 156 had both treatments and 40 had no adjuvant medical therapy.

## Results

### Oncological events

We have observed 30 cases of true recurrences (2.4%). The median time of the appearance of local recurrences was 26.7 months from the operation. More than 70% were detected by radiological examination (mammography, ultrasound and RM).

These 30 patients were either given a second breast-conserving operation (12 cases) or a total mastectomy (18 cases). Twenty-eight patients are well without evidence of disease. One patient developed multiple metastases two years after the second operation and one patient developed liver metastases. Both patients were stabilized after systemic treatment.

There were 11 (0.9%) second primary carcinomas in other quadrants. Eight cases had a second breast resection and 3 underwent total mastectomy. One case developed lung metastases and died from PD and a second died from other causes.

Twenty patients (1.6%) developed distant metastases as first event (Table 3): none of them had signs of local relapse. Altogether eight (0.6%) patients died from breast carcinoma and ten (0.8%) died from other causes.

### Local side effects

#### Immediate post-operative events

Forty-five patients (3.6%) developed acute haematoma, and 15 (1.2%) had post-operative infections in the treated portion of the breast (Table 4).

### Fibrosis

Six patients (0.5%) who received 21 Gy developed severe fibrosis, one of them associated with post-surgical haematoma. An additional 40 (3.2%) patients suffered from mild fibrosis; the development of the fibrosis was progressive during the first months after surgery, reaching a maximum at 12 months, remained stable for another 6–12 months, and within 36 months from surgery slowly regressed. At the time of publication, five patients still have clinical evidence of mild fibrosis in the treated area. Fourteen patients experienced moderate skin retraction.

### Liponecrosis

A limited number of patients experienced a mild post-operative complication, which we defined as ‘liponecrosis’. A localized collection of brown fluid with skin erythema, with no sign of infection, was the clinical manifestation of this complication. We observed 58 (4.7%) cases of liponecrosis 2–4 weeks after surgery. This complication was resolved with simple clinical care and, in one case, requested surgical curettage of the necrotic area. In another case, the surgical scar spontaneously opened due to the fluid pressure, but did not need curettage, and repair of the breech was achieved after two weeks.

Liponecrosis involved mainly patients with very fat breasts; the age range was 38–79 years (median age 60): 30 patients were younger than 60 years, 20 were between 61 and 70, and 8 were older than 70. This complication appears to be more frequent in patients with a higher proportion of fat tissue in the breast.

## Discussion

In the field of radiation therapy for breast cancer following conservative surgery, the question whether the entire breast needs to be irradiated or whether it is sufficient to treat a more limited volume of tissue surrounding the tumour bed (PBI) has become a controversial issue. Recent data show that 80% of local recurrences occur in the scar area (true recurrences), while the remaining 20% occur in other quadrants (new primary carcinomas) [11–13]. Some interesting studies have been published, which aimed at testing the impact of partial breast irradiation on breast cancer treatment; different radiotherapy techniques were used for these studies with controversial results. An early trial at the Christie Hospital showed that whole breast irradiation was superior compared to partial breast irradiation in local control after breast-conserving surgery (10% local recurrences compared to 19.5%, respectively) [14]. On the contrary, Reitsamer [15], in a non-randomized study, showed that the boost given with intra-operative electron radiotherapy is superior to the external breast electron boost irradiation. Partial breast irradiation can be delivered also with interstitial brachytherapy [16–18], and more recently by external sources with three-dimensional radiotherapy (3D-RT) and intensity modulated radiotherapy (IMRT) [19–20].

A report by a study group convened by the National Cancer Institute in 2002 [1] concluded that partial breast irradiation is an interesting new development in breast cancer radiotherapy and deserves to be encouraged.

IORT gives way to a second controversial issue. Intra-operative radiotherapy was first applied nearly a century ago [21] and was developed, with interesting results, in the subsequent decades; mainly to treat abdominal carcinomas or sarcomas, locally advanced or inoperable [22]. The main limitation of the procedure is the logistical problem of transferring the sleeping patient from the surgical theatre to the radiotherapy department, a complicated manoeuvre not easily acceptable to surgeons. The recent appearance of mobile linear accelerators, as a result of new advances of technological research, has overcome these difficulties.

A new, refined method for intra-operative radiotherapy is ‘intrabeam’, which is used in the TARGIT trial. Intrabeam is a miniature electron beam-driven x-ray source that provides a point source of low-energy x-rays (50 kV maximum) at the tip of a 3.2-mm-diameter tube [23]. A phase III randomized trial comparing single-dose intra-operative radiotherapy targeted to the tumour bed to conventional external radiotherapy in early breast carcinoma is presently in progress [24].

We decided some ten years ago to combine the two procedures (PBI and IORT), considering that the single session procedure instead of the conventional six-week course of whole breast irradiation, would substantially ease the difficulties of those women who have to contend with long waiting lists for RT or live far away from a radiotherapy centre. Equally important is that this simpler and quicker treatment will cost much less than conventional RT for breast cancer patients.

**Figure 2:**
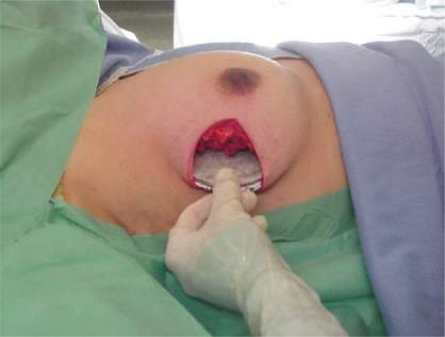
Thoracic wall protection

The ELIOT is a promising feature in breast conservation; the reduction of the radiation field makes the exposure of normal tissues dramatically lower (Figure 2), and the shortening of the radiation course from 5–6 weeks to one session is extremely positive in terms of patients’ quality of life. For this reason, we included assessment of quality of life as a routine part of the ELIOT procedure.

We began clinical research on ELIOT in 1999. The first task was to estimate the single dose of electrons biologically equivalent to standard fractionated radiotherapy for breast cancer. To do this, we used the linear-quadratic surviving fraction model, otherwise known as multi-target surviving fraction model, which indicated that a single dose in the range 20–22 Gy is equivalent to 58–60 Gy delivered in 2 Gy daily fractions, five days a week over six weeks (i.e. the dose required to control microscopic residual disease after breast resection).

Initially, we began with intra-operative doses lower than this level and then increased them. We studied dose levels of 10,15,17,19 and 21 Gy [24–28].

A randomized trial that started on 20 November 2000 is enrolling patients older than 48 years affected by unifocal breast carcinoma with maximum diameter 2.5 cm. Patients receive breast-conserving surgery and are randomized during the operation for ELIOT 21 Gy or external fractionated conventional radiotherapy (50 Gy whole breast and 10 Gy boost to tumour bed). As of November 2007, 1297 patients have been enlisted into the trial: 649 patients received conventional radiotherapy and 648 received ELIOT. We are still recruiting patients, and the follow-up of treated patients is ongoing. As previously mentioned, the 1246 patients described in the present paper are patients who either did not chose to enter the trial or were ineligible.

The toxicity of ELIOT is low; we had just six cases of severe fibrosis that resolved spontaneously within three years of their observation. A further 40 (3.2%) cases of mild fibrosis did not cause serious cosmetic impairment. Fourteen cases of skin retraction were related both to ELIOT and to poor surgical remodelling of the breast. The 58 cases of liponecrosis represent an issue we want to clarify with further follow-up; this non-severe complication seems unrelated to post-operative infection and involved mainly patients with breast tissue largely represented by fat.

A possible contra-indication to the use of ELIOT was the clinical involvement of axillary lymph nodes. We believe that there are no logical reasons for this position, as a primary carcinoma, even with axillary involvement, may be of very limited dimension and therefore may be resolved with conserving surgery and local radiotherapy.

The patients’ quality of life has not been negatively affected according to our routine measurements. In fact, patients usually do not even realize that they underwent radiotherapy during surgery.

The cosmetic results were good in the majority of the patients both as evaluated by the patients themselves and by the doctors. Among the many advantages of ELIOT, we underline the following:

1. The skin remains intact and any possible plastic surgery operation may be easily conducted.
2. Patients with breast carcinoma who had in the past received radiotherapy on the whole breast, either for a previous carcinoma conservatively treated, or for a malignant lymphoma, are easily treated with breast conservation and ELIOT. Those cases, at the appearance of a new primary breast carcinoma, are in many centres treated with mastectomy, as breast conservation may appear problematic, as post-operative radiotherapy cannot be delivered.
3. The availability of the radiotherapy machine during surgery allows additional doses of radiotherapy to be given if needed for specific reasons. For instance, in a few cases where the carcinoma was very close to the pectoralis muscle, a specific boost in that area was delivered.
4. In cases when two primary carcinomas are present, it is easy to perform two separate resections followed by ELIOT in both quadrants.
5. The major advantage remains the complete change of life for patients living in small villages, in the mountains and in the islands far away from radiotherapy centres who very often decide on a mastectomy, even for a very tiny carcinoma, due to enormous problem of undergoing daily post-surgical radiotherapy at these far away centres.
6. An additional advantage of ELIOT is that there is no delay in administering RT in cases that need adjuvant anthracyclines. There is evidence that the delay might increase the risk of local recurrences [30–33].
7. Finally, the complete radioprotection will abolish the side effects in the lung, and in the contralateral mammary gland of conventional whole breast radiotherapy.

One area of concern in the use of ELIOT is the management of positive surgical margins as positivity is discovered at the final histology, a few days after surgery and intra-operative radiotherapy. The adoption of an extensive breast resection as a standard procedure in breast-conserving surgery keeps the incidence of positive surgical margins to very low rates. Moreover, data from different studies show that margin positivity did not influence the rate of local recurrences if effective radiotherapy is delivered [34,35].

We had five cases with positive margins (two with *in situ* neoplasia) that were not re-resected. All five cases have no signs of recurrences 3.9 (1.2–28.1) months after treatment.

## Conclusions

**Figure 3:**
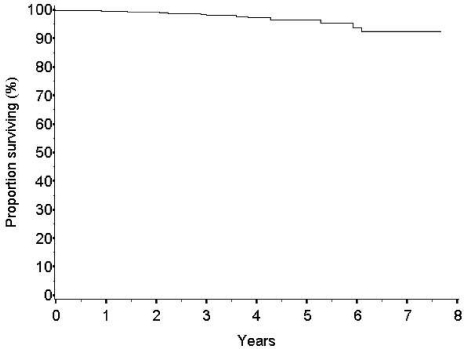
Overall survival

The survival rate at five years is high (96.5%) (Figure 3). The number of breast-cancer-related events observed is low (24 cases, 1.9%). The frequency of local recurrences appears to be similar to that of patients treated with conventional conservative surgery and whole breast radiotherapy. The rate of new primary carcinomas in other quadrants is expected to be somewhat higher after ELIOT than after whole breast radiotherapy but at present is equally low (16 cases, 1.3%).

Our results confirmed the positive impact of ELIOT on patients’ quality of life: ELIOT is feasible and well accepted. We are waiting for the long-term results on local control from the ongoing randomized trial in progress at our institute to decide whether to adopt the technique in daily (as standard) practice. However, as the data from the present large series are reassuring (97% of local control and 98.8% survival at five years) we believe that, at least for women living far from radiotherapy centres and with minimal risk of local recurrence (age > 50 and primary carcinoma <1.5 cm.), the ELIOT treatment might be considered an option, provided that the patient consents to the proposal.

## Figures and Tables

**Table 1:**
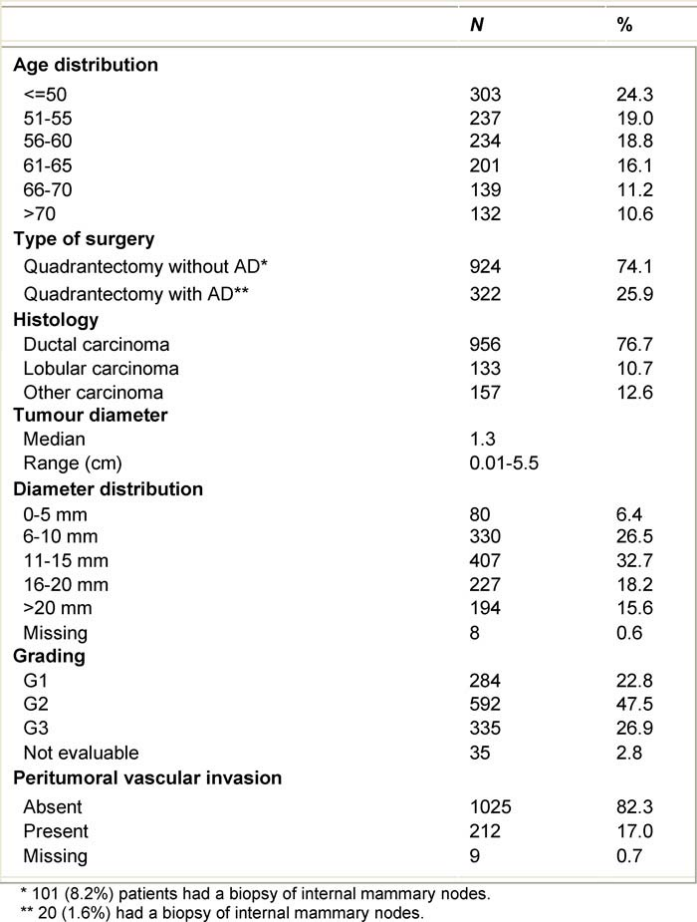
Characteristics of the 1246 ELIOT patients

**Table 2:**
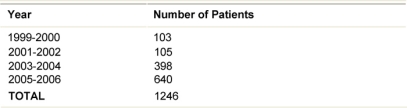
ELIOT patients July 1999-December 2006

**Table 3:**
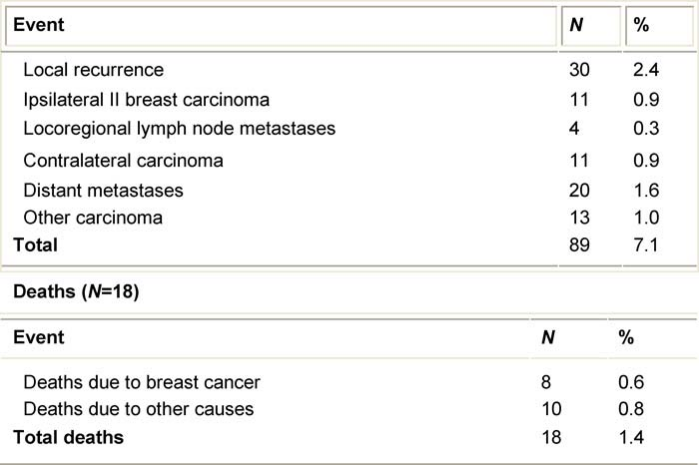
First unfavourable event (89 cases)

**Table 4:**
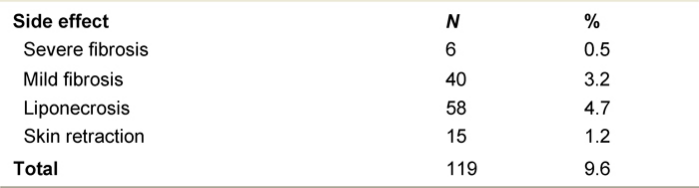
Side effects among 1246 patients
